# Sintilimab plus docetaxel as second-line therapy of advanced non-small cell lung cancer without targetable mutations: a phase II efficacy and biomarker study

**DOI:** 10.1186/s12885-022-10045-0

**Published:** 2022-09-05

**Authors:** Yongchang Zhang, Lianxi Song, Liang Zeng, Yi Xiong, Li Liu, Chunhua Zhou, Haiyan Yang, Zhan Wang, Qing Xia, Wenjuan Jiang, Qinqin Xu, Nong Yang

**Affiliations:** 1 Department of Medical Oncology, Lung Cancer and Gastrointestinal Unit, Hunan Cancer Hospital/The Affiliated Cancer Hospital of Xiangya School of Medicine, Central South University, Changsha, 410013 China; 2 Graduate Collaborative Training Base of Hunan Cancer Hospital, Hengyang Medical School, University of South China, Hengyang, 421001 Hunan China; 3 Department of Medical Oncology, Yiyang Central Hospital, Yiyang, 413000 China; 4 State Key Laboratory for Oncogenes and Related Genes, Shanghai Cancer Institute, Renji Hospital, Department of Oncology, Shanghai Jiao Tong University School of Medicine, Shanghai, China; 5 Department of Medical Oncology, Qinghai Provincial People’s Hospital, Xining, 810000 China

**Keywords:** Sintilimab plus docetaxel, NSCLC, CTC-PD-L1, Multiplex immunofluorescence

## Abstract

**Background:**

Single-agent immunotherapy is currently the recommended second-line therapy for patients with advanced non–small cell lung cancer (NSCLC) without targetable mutations; however, the objective response rate (ORR) remains low. This phase II study evaluated the efficacy of the combination therapy of sintilimab plus docetaxel and explored potential biomarkers for efficacy prediction.

**Methods:**

Thirty patients with NSCLC without targetable mutations whose disease progressed from first-line platinum-based chemotherapy from October 2019 to December 2020 were enrolled in this single-arm, single-center, phase II trial. Sintilimab (200 mg) and docetaxel (75 mg/m^2^) were administered every 3 weeks until progression. The primary endpoint was ORR. Secondary endpoints included progression-free survival (PFS), overall survival (OS), and safety. Biomarker analyses of blood and tissue samples were also performed.

**Results:**

Among 30 patients, 11 patients had partial response, resulting in an ORR of 36.7%. The median PFS was 5.0 months (95%CI: 3.9–6.1) and OS was 13.4 months (95%CI: 5.6–21.2). The most common immune-related adverse event of any grade was hepatitis, observed in 23.3% (7/30) of patients. Treatment-emergent adverse events were manageable. Patients detected with high PD-L1 expression in circulating tumor cells (cutoff value ≥32.5% based on the median CTC-PD-L1 expression) achieved significantly higher ORR (60% versus 13.3%, *p* = 0.021) and significantly longer median PFS (6.0 versus 3.5 months, *p* = 0.011) and median OS (15.8 versus 9.0 months, *p* = 0.038) than those with low CTC-PD-L1 level. Patients detected with PD-L1 < 1% and CD8 ≥ 1% expression from their baseline tissue samples had significantly higher ORR (83.3% versus 12.5%, *p* = 0.026) but similar PFS (*p* = 0.62) and OS (*p* = 0.15).

**Conclusion:**

This study demonstrated the effectiveness and safety of sintilimab plus docetaxel as a second-line treatment of NSCLC without targetable mutations after progression from first-line platinum-based chemotherapy.

**Trial registration:**

This study was registered in the Clinical trials registry with ClinicalTrials.gov Identifier NCT03798743 (SUCCESS).

**Supplementary Information:**

The online version contains supplementary material available at 10.1186/s12885-022-10045-0.

## Highlights

• Survival outcomes of patients with NSCLC without targetable mutations remain depressing.

• Sintilimab plus docetaxel as second-line therapy improves progression-free survival outcomes.

• Sintilimab plus docetaxel is safe and well-tolerated.

• Patients detected with high PD-L1 expression in circulating tumor cells (≥32.5%, median level in 30 patients) achieved significantly higher ORR, PFS and OS.

• Patients detected with PD-L1 < 1% and CD8 ≥ 1% expression from their baseline tissue samples had significantly higher ORR (*p* = 0.026).

**Supplementary Information:**

The online version contains supplementary material available at 10.1186/s12885-022-10045-0.

## Background

The development of lung cancer is determined not only by cancer cell genomics and specific molecular alterations, but also by the interplay between cancer cells and the tumor microenvironment, especially the immune system [1, 2]. The PD-1/PD-L1 immune checkpoint axis was identified as a co-stimulatory pathway that can negatively regulate T cell activation, which leads to tumor cells evading the autoimmune response. The escape of tumor cells from the immune system is important for the occurrence, development, recurrence, and metastasis of lung cancer [1, 3]. The median overall survival (OS) and 5-year survival rates have historically been poor for patients with non-small-cell lung cancer (NSCLC). In the past decade, a number of immune checkpoint inhibitors were approved by the US Food and Drug Administration (FDA) and other regulatory agencies, which revolutionized the therapeutic landscape of lung cancer harboring no actionable mutation [4].

Sintilimab is a novel anti-PD-1 monoclonal antibody (mAb), which exerts its antitumor activity through blocking the interaction between PD-1 and PD-L1 and PD-L2 [5, 6]. Sintilimab’s molecular mechanism of action and pharmacokinetic profile is similar to nivolumab and pembrolizumab [5, 6]. The first in-human clinical trial of sintilimab demonstrated its safety and promising clinical efficacy in advanced solid tumors, including hepatocellular carcinoma and neuroendocrine tumor [7]. Sintilimab was approved by the National Medical Products Administration (NMPA) of China in December 2018 for treating patients with relapsed or refractory classical Hodgkin’s lymphoma [8]. Simultaneously, sintilimab is being investigated in various phases of clinical studies in China as single agent or in combination with other drugs for treating patients with solid tumors, including advanced NSCLC [6, 9–11]. Compared with other well-studied PD-1 inhibitors nivolumab and pembrolizumab, sintilimab is equally effective and safe in the treatment of various cancers and is available at a much lower cost [6], which makes it an attractive treatment regimen.

This study reports the results of the phase II study investigating the safety and efficacy of sintilimab plus docetaxel combination regimen as second-line therapy of patients with advanced NSCLC without actionable mutation after progressing from first-line chemotherapy (SUCCESS trial; NCT03798743). Moreover, potential predictive biomarkers of response to this combination therapy were also explored by performing comprehensive analyses of peripheral blood circulating tumor cells (CTCs) and PD-L1 detection on CTCs, and multiplexed immunohistochemistry (mIHC) of baseline samples of patients enrolled in this study.

## Methods

### Patient selection

Patients deemed eligible to receive the study treatment combination had the following features: 1. ≥ 18 years of age; 2. histologically confirmed locally advanced or metastatic NSCLC without activating mutations in *EGFR*, *ALK*, and *ROS1*; 3. received prior first-line platinum-containing chemotherapy. Patients who received concurrent bevacizumab therapy (7.5 mg/kg intravenously) to either address pleural or pericardial effusion or brain metastasis or at the physician’s discretion were included; 4. received radiological evaluation and confirmed to progress from first-line chemotherapy according to Response Evaluation Criteria in Solid Tumors (RECIST) version 1.1 [12]; 5. at least one measurable lesion; 6. an Eastern Cooperative Oncology Group (ECOG) performance status (PS) of 0 or 1; and 7. adequate bone marrow, liver, renal, and blood clotting function. Disease staging of lung cancer was based on the eighth edition of the tumor node metastasis classification of lung cancer [13, 14]. Patients were excluded if having any of the following features: 1. histologically confirmed small cell lung cancer; 2. participating in clinical research trial or being treated with immune checkpoint inhibitors; and 3. known to have severe autoimmune disease or any syndrome that required systemic steroids or other immunosuppressive agents. Patients with asymptomatic untreated brain metastases or with stable symptoms after treatment were required to meet the inclusion criteria, in which the measurable target lesions should be outside the central nervous system; without midbrain, pons, medulla, cerebellum, and spinal cord metastasis. Patients with previously treated brain metastases should be clinically stable for at least 2 weeks and had not received steroids within 3 days before the first dose of the study treatment. The patients enrolled in this study must provide 20 slides of formalin-fixed paraffin-embedded (FFPE) tissue microsections, with at least 5 slides with adequate tumor content for fluorescent mIHC staining. The study was registered on ClinicalTrials.gov (NCT03798743), with the study protocol approved by the Hunan Cancer Hospital Institutional Review Board Committee (2019YYQ -SSB-019). The study was performed in accordance with the Declaration of Helsinki and the International Council for Harmonisation of Technical Requirements for Pharmaceuticals for Human Use (ICH) Good Clinical Practice guidelines. Before inclusion in the trial, all patients provided written informed consent. The enrolled patients were not compensated for participation.

### Study design and procedures

This phase II, single-arm, single-center, prospective, nonrandomized open-label clinical trial was performed at Hunan Cancer Hospital in Hunan, China. The study began recruitment from October 2019 until December 2020. The data cutoff date was June 30, 2021. For this exploratory study, a fixed sample size of 30 patients was set. Biomarker analyses were performed on blood and tissue samples collected from all patients before administration of the regimen (at baseline). Biomarkers analyzed for this study included the detection of circulating tumor cells (CTCs) or CTC-based PD-L1 expression, and various tissue-based immune markers. Patients were administered sintilimab and docetaxel at standard recommended doses as follows: intravenous drip sintilimab 200 mg and docetaxel 75 mg/m^2^ every 3 weeks for up to 24 months or until disease progression (with the target lesions evaluated periodically using RECIST version 1.1 [12]) or unacceptable toxicity. Dose adjustments were determined by the principal investigator. The primary endpoint was objective response rate (ORR) determined by internal radiology review (IRR). Secondary endpoints were progression-free survival (PFS), overall survival (OS), and safety. Biomarkers associated with response to the combination therapy was exploratory. ORR was defined as the proportion of patients who achieved complete response (CR) or partial response (PR). PFS was defined by the time (in months) from receiving the combination therapy until disease progression or cancer-related death. OS was defined by the time (in months) from receiving the combination therapy until death or last follow-up.

### Study assessments

Tumor assessments were performed before treatment with sintilimab and docetaxel. Treatment evaluation was performed every 6 weeks (±7 days) for the first 12 months (48 weeks), and then every 12 weeks (±14 days) until disease progression by IRR or treatment discontinuation. Systemic response was assessed according to RECIST (version 1.1) [12]. Adverse events (AEs) were classified and graded every cycle of treatment according to National Cancer Institute Common Terminology Criteria for Adverse Events (version 4.03) [15].

### Immunomagnetic bead preparation for CTC isolation and PD-L1 staining

IsoFlux system (Fluxion, South San Francisco, CA, USA) was used for CTC isolation, which utilizes immunomagnetic beads that target the selected antigens in the cancer cell surface and enriches CTCs in blood samples. The original protocol for CTC enrichment using the IsoFlux system was modified to replace the IsoFlux beads with CELLection Epithelial Enrich Dynabeads (Thermo Fisher Scientific, Waltham, MA, USA). Dynabeads coated with human anti-mouse IgG were utilized to enrich cells. Then, it was incubated at room temperature with an anti-epithelial cell adhesion molecule (EpCAM) antibody (Clone Ber-EP4, Abcam; 0.02 μg antibody/μL bead suspension). After incubation, the beads were washed thrice with phosphate-buffered saline supplemented with 0.1% bovine serum albumin and were stored at 4 °C. All the CTCs were stained with PD-L1 following standard protocol from the manufacturer.

### PD-L1 expression and fluorescent mIHC

All the baseline FFPE tissue samples were stained with 22C3 (Dako Omnis Agilent Technologies, Santa Clara, CA, USA) for evaluating PD-L1 expression. Fluorescent mIHC was performed as previously described in our report [16]. Tissue biopsy and postoperative surgical tissue samples collected before receiving the study treatment were processed as FFPE blocks and then sliced into 4-μm tissue sections, which were mounted on microscope slides. Staining of the slides was performed using the Opal 7-color IHC kit (Catalog number: NEL797B001KT; PerkinElmer, Massachusetts, USA). The 2 immune markers evaluated included CD8 (Catalog number: ZA-0508, clone SP16; Zsbio; 1:100) and PD-L1 (Catalog number: CST13684, clone E1L3N, Cell Signaling Technology, 1:100). For CD8 and PD-L1 expression, tumor proportion score of < 1% was considered negative [17]. The study cohort was stratified into four subgroups based on their pre-treatment tissue PD-L1 expression (≥1 and < 1%) and CD8 expression (≥1 and < 1%) status as PD-L1-positive (≥1%)/CD8-positive (≥1%), PD-L1-negative (< 1%)/CD8-positive (≥1%), PD-L1-positive (≥1%)/CD8-negative (< 1%), and PD-L1-negative (< 1%)/CD8-negative (< 1%). Markers were identified and quantified by fluorescent mIHC. Briefly, the slides were deparaffinized, rehydrated, and washed in tap water before epitope retrieval/microwave treatment. Endogenous peroxidase activity was blocked using Antibody Diluent/Block (Catalog number: 72424205; PerkinElmer, Massachusetts, USA). One antigen required one round of labeling, including primary antibody incubation, secondary antibody incubation, and visualization with tyramide signal amplification (TSA), followed by labeling with the next antibody. Counterstaining was performed using hematoxylin. Slides were scanned using PerkinElmer Vectra (Vectra 3.0.5; PerkinElmer, Massachusetts, USA). The percentage of positively stained cells among all nucleated cells was evaluated.

### Statistical analyses

DOR was summarized by the Kaplan-Meier method and descriptive statistics. ORR and DCR were evaluated, and the 95% confidence intervals (CIs) were calculated using binomial distribution. The Kaplan-Meier method was used to estimate median PFS and OS. The Cox proportional hazards model was used for multivariable survival analysis. Hazard ratios (HRs) were computed by the Schoenfeld residuals and 95% CIs were calculated to determine the survival difference. Statistical significance was defined as two-sided and *p* < 0.05, unless stated otherwise. All analyses were done using either SPSS software (version 24) or R Studio (version 1.1.383).

## Results

### Patient demographics and baseline characteristics

**Fig. 1:**
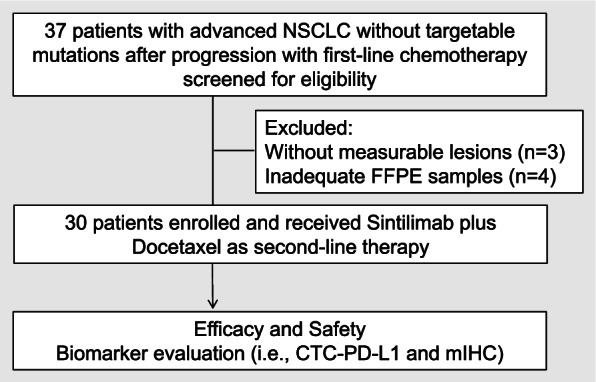
Flow diagram of the study design

From October 2019 through December 2020, 37 patients with NSCLC without targetable mutations after progressing from first-line chemotherapy were screened for eligibility (Fig. 1). A total of 30 patients were included in the study and received sintilimab plus docetaxel as second-line therapy. Among them, 22 (73.3%) were males, and 14 (46.7%) were never smokers. The most frequent histologic diagnosis was lung squamous cell carcinoma, accounting for 16 (53.3%) patients. All 30 patients had no targetable mutations. Furthermore, among the 30 patients, five (16.7%) patients had PD-L1 expression of ≥50%, 13 (43.3%) patients had PD-L1 between 1 and 49%, and 12 (40%) patients had PD-L1 < 1%. The most common regimen received for first-line therapy was carboplatin and paclitaxel, which were received by 15 patients (50%). No patient received immunotherapy as first-line therapy. Table 1 lists the baseline clinicopathological characteristics of the cohort.

**Table 1:** Baseline clinical characteristics of the study cohort and best response to combination therapy

Characteristics	Total, No. (%)
No. of patients	30
Age, years	
Median	56
Range	48–69
Sex	
Male	22 (73.4)
Female	8 (26.6)
Smoking history	
Never smoker	14 (46.7)
Former smoker	16 (53.3)
Histology	
Adenocarcinoma	16 (53.3)
Squamous carcinoma	14 (46.7)
Eastern Cooperative Oncology Group performance status	
0–1	30 (100)
≥ 2	0
Brain metastasis	
Yes	1 (3.3)
No	29 (96.7)
Stage	
IIIa/IIIb	4 (13.4)
IV	26 (86.6)
Best response with sintilimab plus docetaxel	
Complete Response	0
Partial Response	11 (36.7)
Stable Disease	13 (43.3)
Progressive Disease	6 (20)
Objective Response Rate	36.7%

### Efficacy

**Fig. 2:**
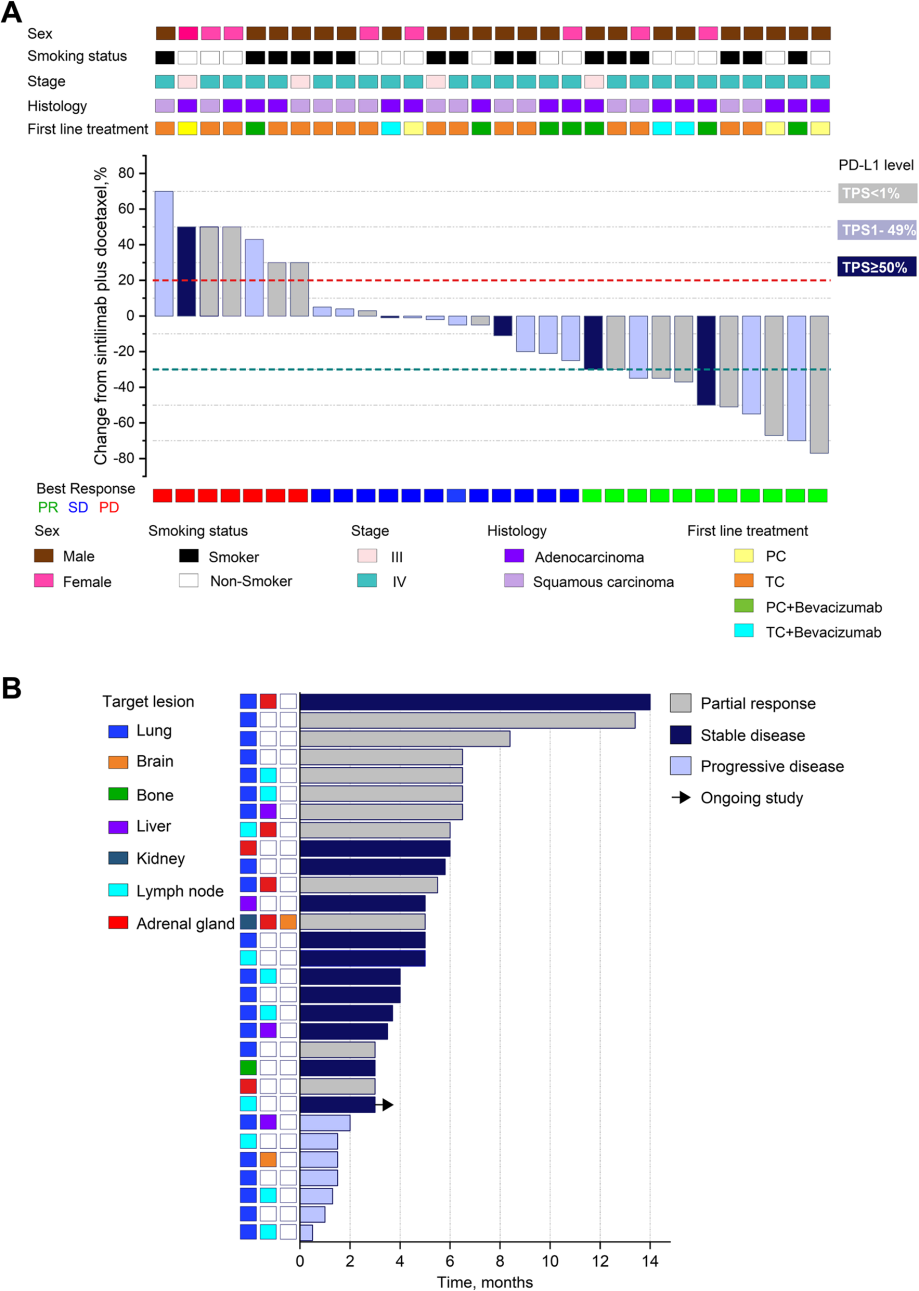
Clinical outcomes associated with second-line sintilimab plus docetaxel therapy. Waterfall plot (**A**) and swimmers plot (**B**) illustrating the best change in tumor size and duration of response of each of the 30 patients who received sintilimab plus docetaxel as second-line therapy. Clinical details and the PD-L1 tumor proportion score (TPS) subgroup of each patient were indicated by different colors

**Fig. 3:**
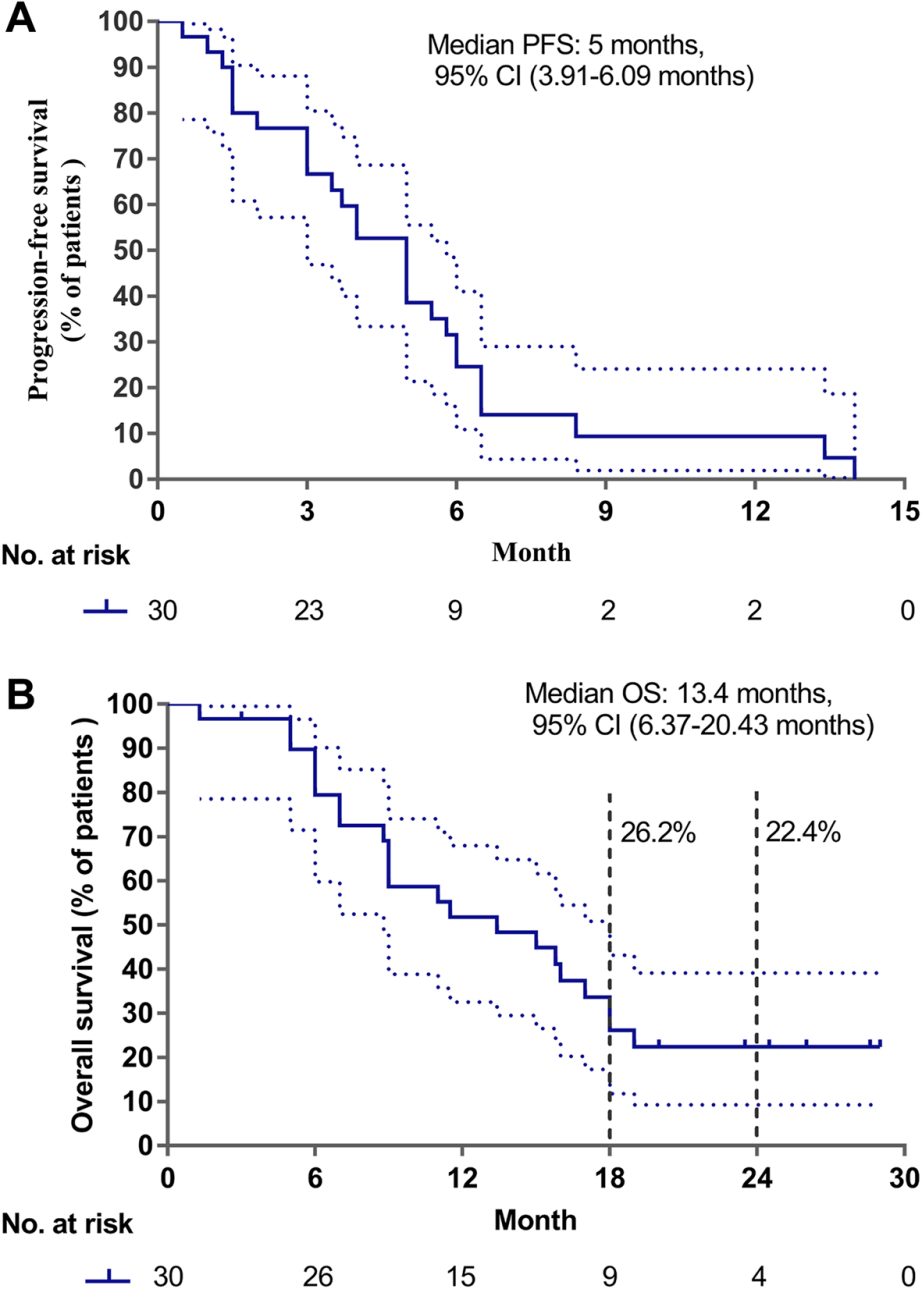
Survival outcomes associated with second-line sintilimab plus docetaxel therapy. Kaplan-Meier plots summarizing the progression-free survival (PFS) (**A**) and overall survival (OS) (**B**) of the 30 patients who received sintilimab plus docetaxel therapy. The dotted lines indicate the 95% confidence intervals (CI). Vertical dotted lines indicate the 18-month and 24-month OS. The risk table below indicates the number of patients analyzed per time point. Tick marks indicate the censored patients

Of the 30 patients who received sintilimab plus docetaxel as the second-line treatment, 11 had PR, 12 had SD, none achieved CR after receiving two cycles of treatment, and seven patients had progressive disease, resulting in an ORR of 36.7% (*n* = 11). Figure 2A and B illustrate the tumor shrinkage in 30 patients. The maximum size reduction of target lesions relative to the baseline was 80% (Fig. 2A). As of the data cutoff date, the median follow-up was 26.5 months. The median PFS was 5.0 months (95% CI: [3.91, 6.09 months]; Fig. 3A) and the median OS was 13.4 months (95% CI: [6.37, 20.43 months]; Fig. 3B). The 18-month OS was 26.2% and the 24-month OS was 22.4% (Fig. 3B).

As indicated in Fig. 2A, 10 patients received bevacizumab concurrently with platinum-based chemotherapy at the first-line setting. Among these 10 patients, the ORR for second-line sintilimab plus docetaxel was 50% (5/10) and was numerically higher than those who did not receive bevacizumab (30%, 6/20); however, the difference did not reach statistical significance (*p* = 0.28).

### Safety

Table S1 tabulates the treatment-related adverse events (TRAEs) observed in our cohort. At the data cutoff date, the median PFS was 5 months (range from 0.5 to 14 months), and a patient (3.3%) was still receiving sintilimab plus docetaxel. Most of TRAEs were grade 1 or 2 (60.0% for chemotherapy and 36.7% for immunotherapy). The most frequently reported chemotherapy-related AEs of any grade were fatigue (20%), vomiting (13.3%), and anemia (10%). Overall, grade 3 or 4 AE related to docetaxel was reported in only a patient (3.3%) who had grade 3 fatigue and subsequently had chemotherapy interruption but continued sintilimab therapy. This patient had progressive disease as best response with a PFS of 1.3 months. The most frequently reported immune-related AEs of any grade were hepatitis (23.3%), hypothyroidism (6.7%), and pneumonitis (6.7%). Grade 3 or 4 events related to sintilimab were reported in three patients (10%) who had hepatitis and subsequently had immunotherapy interruption. Of these three patients, a patient permanently discontinued the regimen, whereas two patients resumed treatment after temporary interruption. The best response was PR for the patient who terminated the combination regimen, and PR and SD, respectively, for the two patients who had temporary interruption and resumed the combination regimen. The toxicity profile of the combination regimen was manageable. No treatment-related death was reported in our study.

### High expression of PD-L1 in CTCs predicts higher ORR, longer PFS and OS with sintilimab and docetaxel therapy

**Fig. 4:**
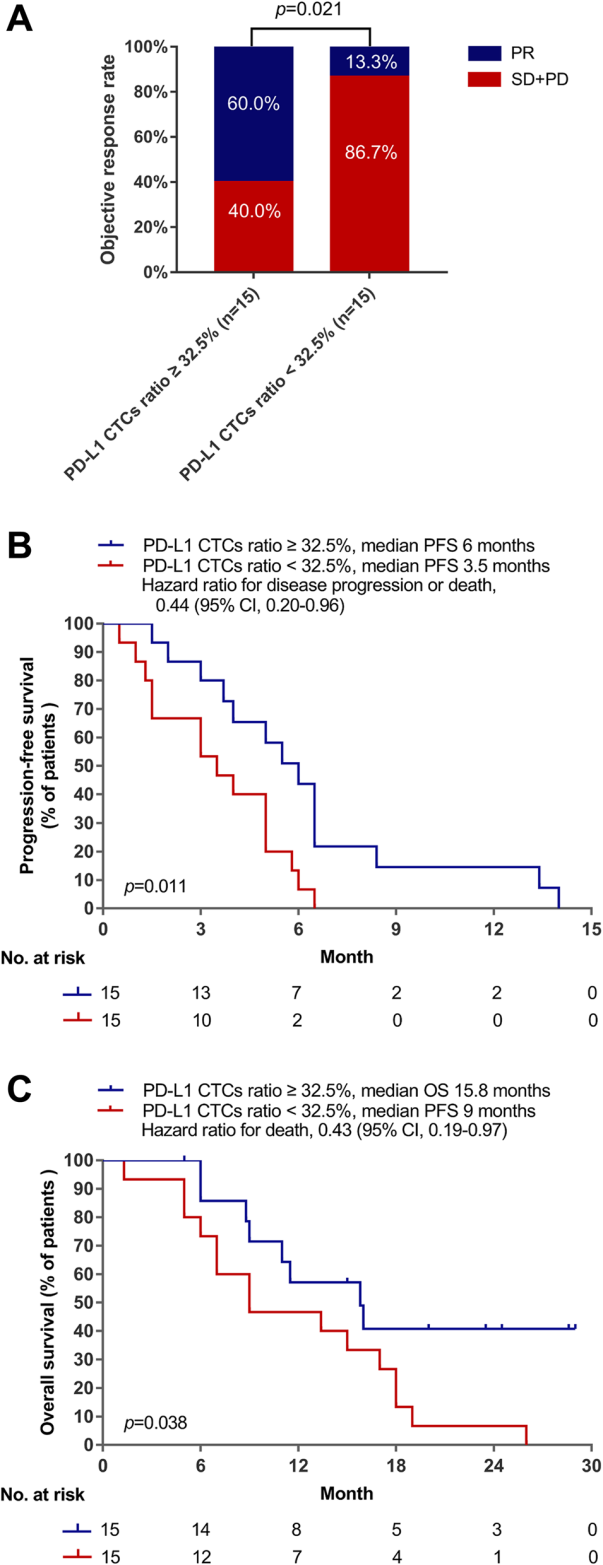
Potential blood-based predictive biomarkers of response to sintilimab plus docetaxel therapy. High PD-L1 expression in circulating tumor cells (CTC) of pre-treatment blood samples was associated with a trend of higher objective response rate (**A**), and longer progression-free survival (PFS) (**B**)

CTCs were isolated from the blood samples collected from all 30 patients before receiving the combination therapy. CTCs were detected at a median of 7.8 CTCs/mL, ranging between 1 and 20 CTCs/mL of whole blood per patient. PD-L1 expression in the CTCs were analyzed. The cohort had a median ratio of PD-L1 expressing CTC of 32.5% (Fig. S1), and was used as the cut-off to categorize the cohort into two subgroups as high or low ratio of PD-L1 expressing CTC. Clinical features were comparable between the two subgroups (Table S2). The subgroup with PD-L1-high expressing CTCs (≥32.5%, *n* = 15) had significantly higher ORR than the subgroup with PD-L1-low expressing CTCs (< 32.5%, *n* = 15) (60% vs. 13.3%; *p* = 0.021; Fig. 4A). Next, we compared the survival outcomes of these two subgroups. Compared with patients with low PD-L1 expression in CTCs, those with high PD-L1 expression in CTCs had significantly longer median PFS (6.0 vs. 3.5 months, *p* = 0.011; HR: 0.44, 95% CI: 0.20–0.96; Fig. 4B) and longer median OS (15.8 versus 9.0 months, *p* = 0.038; HR: 0.43, 95% CI: 0.19–0.97).

### Patients with CD8+/PD-L1- respond better to sintilimab and docetaxel therapy

**Fig. 5:**
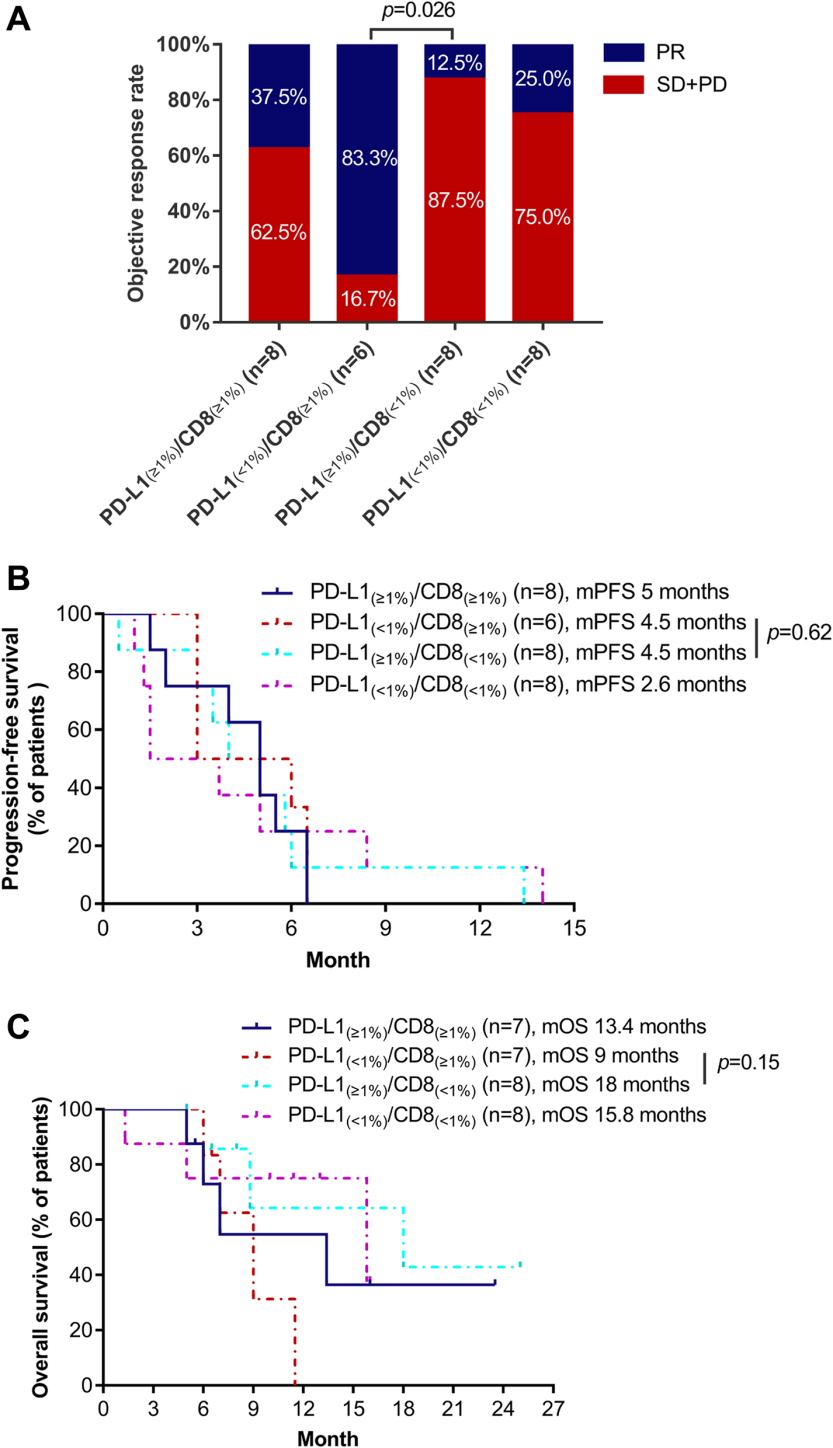
Potential tissue-based predictive biomarkers of response to sintilimab plus docetaxel therapy. Low PD-L1/High-CD8 expression in pre-treatment tissue samples was associated with higher objective response rate (**A**), but no significant difference in progression-free survival (PFS) and overall survival time (**C**)

Among the patients in the cohort, 16 patients had CD8-negative T cells (CD8 < 1%), while 14 patients had CD8-positive T cells (CD8 ≥ 1%). Moreover, 14 patients were PD-L1-negative (PD-L1 TPS < 1%), whereas 16 patients were PD-L1-positive (PD-L1 TPS ≥1%). The baseline clinical features of these subgroups were comparable (Table S3). The study cohort was further stratified into four subgroups based on the positivity of their pre-treatment tissue for PD-L1 expression (≥1 and < 1%) and CD8 expression (≥1 and < 1%). These subgroups had similar baseline clinical characteristics (Table S4). Among the four subgroups, patients with high CD8-positive expression and PD-L1-negative expression (*n* = 6) had significantly higher ORR than those with CD8-negative and PD-L1-positive expression (*n* = 8, ORR: 83.3% vs. 12.5%, *p* = 0.026; Fig. 5A). No significant difference was observed in PFS (*p* = 0.62) and OS (*p* = 0.15) (Fig. 5B and C).

## Discussion

Immune checkpoint inhibitors are widely studied in cancer immunotherapy. Numerous clinical trials were designed to explore various therapeutic approaches that incorporate immunotherapy in the treatment algorithm of various cancers, including NSCLC. The mechanism of anti-cancer activity of chemotherapy combined with immunotherapy is through the induction of immunogenic cell death as part of its intended therapeutic effect and also through the disruption of molecular signals that tumors use to evade immune recognition. In squamous NSCLC (Keynote-407 trial) and non-squamous NSCLC (Keynote-189 trial), first-line combination therapies that integrate immunotherapies with chemotherapy have shown remarkable clinical efficacy in patients with no sensitizing *EGFR* or *ALK* mutations [18].

Currently, immunotherapy is the standard first-line therapy for patients with advanced NSCLC without actionable mutations owing to its survival benefit [19]. Various phase III clinical trials, including ORIENT 11/12 [10, 11, 20], RATIONALE 304/307 [21, 22], CameL/CameL-sq [23, 24], have shown improved ORR, PFS, and OS outcomes with first-line use of the combination of chemotherapy and PD-1 inhibitors such as sintilimab, tislelizumab, and camrelizumab for treating Chinese patients with advanced lung adenocarcinoma or squamous cell carcinoma. In China, the annual cost of immunochemotherapy combination amount to between 30,000–40,000 Chinese yuan. However, due to cost restrictions and limited indications of newer drugs or treatment combinations, these drugs are still not widely available in some hospitals and are not covered by the current national health insurance reimbursement policy. Despite health insurance coverage in some urban hospitals, 50–60% of patients with advanced lung cancer being treated in county-level or city-level public hospitals do not have access to immunotherapy. Moreover, approximately 10,000 patients with advanced NSCLC are enrolled in a number of phase III clinical trials on first-line use of PD-1/PD-L1 mABs, including toripalimab (JS001) and AK-105, with 35–50% of patients randomized to the chemotherapy arm unable to benefit from first-line immunotherapy. Hence, many patients are still unable to receive immunotherapy in the first-line setting due to limited access in less developed regions, cost constraints, and patient comorbidities.

The current standard second-line treatment for NSCLC without targetable mutations is either chemotherapy with docetaxel or PD-1 antibody monotherapy; however, the efficacy of these treatment strategies is still limited. Based on the results of CheckMate 017 and CheckMate 057 [25–27], nivolumab was recommended as the second-line treatment regimen for patients with NSCLC without actionable genetic mutations, including *EGFR* and *ALK*, after progressing from first-line platinum-containing chemotherapy [19]. Despite having better survival outcomes than docetaxel alone, the survival outcomes with nivolumab remain depressing, with approximately 3 months of PFS benefit, 5-year PFS rate of 8.0%, and 5-year OS rate of 13.4% for this group of patients [25–27]. There is still limited research data on the efficacy of second-line chemotherapy combined with immunotherapy for patients with advanced NSCLC without targetable mutations. These observations raise the need to explore novel treatment strategies in improving the clinical outcomes of patients with advanced NSCLC without targetable mutations who progressed from first-line chemotherapy. This study reported the efficacy and safety of a domestic PD-1 inhibitor, sintilimab combined with docetaxel for this subset of patients. Furthermore, we also explored potential biomarkers using tissue and blood samples to identify the patients who could benefit from the sintilimab and docetaxel combination therapy.

Our clinical and survival results demonstrate the efficacy and tolerability of the combination of sintilimab and docetaxel as second-line treatment after progression from chemotherapy of patients with advanced NSCLC without targetable mutations. Our findings suggest the potential use of sintilimab and docetaxel combination as an alternative treatment option in this patient population. Our cohort achieved an ORR of 36.7% and median PFS of 5.0 months to second-line treatment with sintilimab and docetaxel. Nivolumab has been recommended as the second-line treatment option for this patient population based on the promising results from CheckMate 017 and CheckMate 057 [25–27]. However, the PFS benefit was only 3 months for this group of patients [25–27]. Previous studies have also shown a low ORR ranging between 9 and 18% for patients who received docetaxel as second-line regimen after progression from first-line platinum-based chemotherapy [25–28]. Thus, an alternative second-line regimen that could improve the clinical and survival outcomes of this patient population after progression from chemotherapy remains an unmet need. The phase 2 randomized PROLUNG study demonstrated a better ORR (42.5% vs 15.8%) and longer PFS (9.5 vs 3.9 months) for patients who received pembrolizumab plus docetaxel (*n* = 40) as compared with those who received docetaxel only (*n* = 38) as second-line regimen following platinum-based chemotherapy and regardless of *EGFR* mutation status and PD-L1 expression status [29]. The improved ORR and prolonged PFS with the combined use of immune checkpoint inhibitor and chemotherapy of the second-line pembrolizumab docetaxel combination reported by the PROLUNG study is largely consistent with the efficacy and safety of sintilimab and docetaxel combination we observed in our cohort. Our results were also consistent with the clinical efficacy and safety of sintilimab plus docetaxel regimen as second-line therapy in Chinese patients with advanced NSCLC reported by Han and colleagues [30]. Their phase 2 study reported an ORR of 32.4%, DCR of 89.2%, PFS of 5.8 months with second-line docetaxel (75 mg/m^2^) plus sintilimab (200 mg) regimen in 40 Chinese patients with advanced NSCLC [30]. Moreover, biomarker analyses suggested that a reduction in blood-based TMB at 6 weeks after treatment initiation was associated with a significantly longer PFS [30].

Although immune checkpoint inhibitor treatment confers clinical benefit to patients with various solid cancers, biomarkers that could predict the patient’s response to these inhibitors remains inconclusive [31]. Tissue-based PD-L1 expression had been considered as one of the predictive biomarkers for immune checkpoint inhibitor therapy; however, PD-L1 expression may vary between primary and metastatic tumors, which could contribute to heterogeneity in treatment responses [32, 33]. CTCs obtained from patients with advanced cancer could reflect the spatial heterogeneity of the tumor cell population from both primary and metastatic tumors [34, 35]. Numerous studies have demonstrated the relationship between high PD-L1 expression on CTCs and the poor prognosis of patients with various solid cancers, including lung cancer [36, 37]. Dynamic changes of PD-L1 expression on CTCs of patients with advanced solid tumors undergoing PD-1 inhibitor therapy were found to be predictive of therapeutic response wherein a higher ratio of high PD-L1-CTC at baseline is positively correlated to disease benefit [38]. Our findings demonstrate that positive PD-L1 expression on CTCs isolated from pre-treatment blood samples of patients was associated with better ORR, longer PFS, and OS with sintilimab plus docetaxel combination therapy. PD-L1 on CTCs can bind to PD-1 expressed on T cells, leading to the immune escape of circulating tumors [35, 39]. The combination of chemotherapy and immune checkpoint inhibitors could synergistically block this process. CTC-PD-L1 expression may serve as a non-invasive method to select the subpopulation of patients who could benefit with such treatment.

Fluorescent multiplex immunohistochemistry (mIHC) is rapidly becoming a routine tool to simultaneously assess and profile multiple markers of the tumor immune microenvironment (TME), including the tumor-infiltrating lymphocyte (TIL) phenotype [40]. Multimodality biomarker assessment and mIHC, were associated with improved performance in predicting response to anti-PD-1/PD-L1 therapy over IHC-based PD-L1 expression status, tumor mutational burden (TMB), or gene expression profiling (GEP) alone [41]. Our findings also support the multimodality strategy of using various sample types in the assessment of various biomarkers such as PD-L1 on CTCs and tissue-based evaluation of the TME. Interestingly, retrospective analysis of blood samples from patients included in the PROLUNG study found that tissue PD-L1 expression was not predictive of response to pembrolizumab plus docetaxel therapy [42]. Contrastingly, their findings found that dynamic PD-L1 expression from extracellular vesicles could stratify the patients who could benefit from this treatment regimen [42]. Non-responders from pembrolizumab plus docetaxel therapy had a significantly increased PD-L1 expression in extracellular vesicles, which was independently associated with shorter PFS and OS [42]. High PD-L1 expression has been associated with poor clinical outcomes and prognosis in NSCLC, which positions it as an attractive therapeutic target [43–45]. However, PD-L1 expression status is an unreliable marker for predicting response to immunotherapy [46]. An approach that classifies tumors into four tumor microenvironment types based on their PD-L1 status and presence/absence of TILs has been proposed and includes the following types: PD-L1 positive with TILs driving adaptive immune resistance (type 1), PD-L1 negative with no TIL indicating immune ignorance (type 2), PD-L1 positive with no TIL indicating intrinsic induction (type 3), and PD-L1 negative with TIL indicating the role of other suppressors in promoting immune tolerance (type IV) [47]. A study has shown that such tumor classification based on PD-1/PD-L1 and CD8+ TILs could reflect clinical outcomes, wherein patients whose adenocarcinomas had high CD8+ TILs and low PD-1/PD-L1 achieved better survival outcomes, whereas those with low CD8+ TILs and high PD-1/PD-L1 had the worse outcomes [48]. Our findings demonstrate that CD8+/PD-L1- tumors had significantly better ORR to sintilimab plus docetaxel therapy than CD8−/PD-L1+ tumors; however, survival outcomes were not statistically significant. CD8 is mainly expressed on the surface of cytotoxic T cells and plays a crucial part in mediating anti-tumor immune response. A systematic review and meta-analysis of 33 studies have demonstrated that high CD8+ T cells in tissue (i.e., intra-tumoral, stromal, or invasive marginal), but not circulating CD8+ T cells are predictive of treatment outcomes and better prognosis with immune checkpoint inhibitors across cancer types (e.g., NSCLC, melanoma, others) or treatment type (i.e., single-agent or combination therapy of immune checkpoint inhibitor) [49]. Further research is warranted to identify effective and robust biomarkers, including biomarkers related to the TME and PD-L1, which could guide the selection of patients who would benefit from anti-PD-1/PD-L1 therapy.

Our study has several limitations. First, our study was conducted in a single institution, which could introduce patient sampling bias. Second, our study only included a very limited sample size, which could contribute to bias in the data interpretation, particularly in subgroup analysis for the survival outcomes and biomarker analyses. Based on the promising efficacy of sintilimab and docetaxel therapy, we plan to extend our clinical study to include a larger cohort from multiple cancer centers to establish our preliminary findings. As the combination of immunochemotherapy enters clinical practice as one of the standard-of-care first-line regimens, it would also be clinically meaningful to explore the efficacy and safety of docetaxel plus immunotherapy combination regimens in patients with advanced NSCLC after progression from platinum-based chemotherapy and immune checkpoint inhibitors. Docetaxel plus antiangiogenic inhibitors such as ramucirumab [50] or nintedanib [51] has been shown to have better efficacy than docetaxel alone as second-line regimen. Contrastingly, the combination of antiangiogenic inhibitors and immune checkpoint inhibitors (eg, bevacizumab plus atezolizumab) has shown promising antitumor activity and acceptable toxicity profile in patients with advanced NSCLC progressing from first-line platinum-containing chemotherapy [52]. Preclinical studies have implicated the enhancement in infiltration and cytotoxic function of CD8+ T cells in mediating the antitumor activity of the combination of antiangiogenic and immune checkpoint inhibitors [53]. Triplet combinations with antiangiogenics, such as the combination of docetaxel, ramucirumab and pembrolizumab [54], could also be appealing in this setting.

In conclusion, our study demonstrated that the combination of sintilimab and docetaxel has promising efficacy and manageable toxicities as second-line treatment of patients with advanced NSCLC without targetable mutations, suggesting this combination regimen as a potential alternative treatment option in this patient population. The blood-based and tissue-based predictive biomarkers could enable the identification of patients who would likely benefit from this combination regimen. Further clinical studies with a larger cohort are required to evaluate the efficacy of this combination regimen and the validity of the predictive biomarkers.

## Supplementary Information

**Additional file 1: Table S1.** Treatment-related adverse events observed from the study cohort (*N* = 30)**. Table S2.** Baseline clinical characteristics of 30 patients with NSCLC stratified according to their PD-L1 expression in circulating tumor cells (CTC PD-L1) ratio using a cutoff value of 32.5%. **Table S3.** Baseline clinical characteristics of 30 patients with NSCLC stratified according to a cutoff value of 1% for CD8 T cell and tissue-based PD-L1 expression. **Table S4.** Baseline clinical characteristics of 30 patients with NSCLC stratified into 4 subgroups according to PD-L1 tumor proportion score and CD8+ T cell using a cutoff value of 1%. **Fig. S1.** The median ratio of PD-L1 expressing circulating tumor cells (CTC).

## Data Availability

The data that support the findings of this study are available from the corresponding author upon reasonable request.
