# Supplementary material for: Sintilimab plus docetaxel as second-line therapy of advanced non-small cell lung cancer without targetable mutations: a phase II efficacy and biomarker study
Source: BMC Cancer. 2022 Sep 5;22:952. doi: 10.1186/s12885-022-10045-0 (PMC9446552; doi:10.1186/s12885-022-10045-0)

**Table S1.** Treatment-related adverse events observed from the study cohort (N=30)

| Adverse Event (AE) | All Grades | Grade 1 | Grade 2 | Grade 3 | Grade 4 |
| --- | --- | --- | --- | --- | --- |
|  | No. (%) | No. (%) | No. (%) | No. (%) | No. (%) |
| Chemotherapy-related AEs | | | | | |
| Fatigue | 6(20%) | 3(10%) | 2(6.7%) | 1(3.3%) | 0 |
| Anemia | 3(10%) | 2(6.7%) | 1(3.3%) | 0 | 0 |
| Nausea | 1(3.3%) | 1(3.3%) | 0 | 0 | 0 |
| Neutropenia | 2(6.7%) | 1(3%) | 1(3.3%) | 0 | 0 |
| Vomiting | 4(13.3%) | 2(6.7%) | 2(6.7%) | 0 | 0 |
| Thrombocytopenia | 2(6.7%) | 0 | 2(6.7%) | 0 | 0 |
| Immune-related AEs | | | | | |
| Hypothyroidism | 2(6.7%) | 1(3.3%) | 1(3.3%) | 0 | 0 |
| Pneumonitis | 2(6.7%) | 0 | 2(6.7%) | 0 | 0 |
| Hepatitis | 7(23.3%) | 3(10%) | 1(3.3%) | 3(10%) | 0 |

**Table S2**. Baseline clinical characteristics of 30 patients with NSCLC stratified according to their PD-L1 expression in circulating tumor cells (CTC PD-L1) ratio using a cutoff value of 32.5%.

| Characteristics | | PD-L1 CTC ratio | | *p* |
| --- | --- | --- | --- | --- |
|  |  | <32.5% (n=15) | ≥32.5% (n=15) |  |
| Sex | Female | 5 (33.3%) | 3 (20%) | 0.680 |
|  | Male | 10 (66.7%) | 12 (80%) |  |
| Age | <56 years | 8 (53.3%) | 7 (46.7%) | 1.000 |
|  | ≥56 years | 7 (46.7%) | 8 (53.3%) |  |
| Smoking history | Former smoker | 8 (53.3%) | 8 (53.3%) | 1.000 |
|  | Never smoker | 7 (46.7%) | 7 (46.7%) |  |
| ECOG PS score | 0-1 | 15(100%) | 15(100%) | 1.000 |
|  | ≥2 | 0 | 0 |  |
| Stage | IIIa/IIIb | 3 (20%) | 1 (6.7%) | 0.591 |
|  | IV | 12 (80%) | 14 (93.3%) |  |
| Histology | Adenocarcinoma | 6 (40%) | 10 (66.7%) | 0.272 |
|  | Squamous carcinoma | 9 (60%) | 5 (33.3%) |  |

**Table S3**. Baseline clinical characteristics of 30 patients with NSCLC stratified according to a cutoff value of 1% for CD8 T cell and tissue-based PD-L1 expression.

| Characteristics | | CD8 T cell | | *p* | PD-L1 level | | *p* |
| --- | --- | --- | --- | --- | --- | --- | --- |
|  |  | <1% (n=16) | ≥1% (n=14) |  | <1% (n=14) | ≥1% (n=16) |  |
| Sex | Female | 5 (31.2%) | 3 (21.4%) | 0.847 | 3 (21.4%) | 5 (31.2%) | 0.847 |
|  | Male | 11 (68.8%) | 11 (78.6%) |  | 11 (78.6%) | 11 (68.8%) |  |
| Age | <56 years | 7 (43.8%) | 8 (57.1%) | 0.714 | 7 (50%) | 8 (50%) | 1 |
|  | ≥56 years | 9 (56.2%) | 6 (42.9%) |  | 7 (50%) | 8 (50%) |  |
| Smoking history | Former smoker | 7 (43.8%) | 9 (64.3%) | 0.448 | 7 (50%) | 9 (56.2%) | 1 |
|  | Never smoker | 9 (56.2%) | 5 (35.7%) |  | 7 (50%) | 7 (43.8%) |  |
| ECOG PS score | 0-1 | 16(100%) | 14(100%) | 1 | 14(100%) | 16(100%) | 1 |
|  | ≥2 | 0 | 0 |  | 0 | 0 |  |
| Stage | IIIa/IIIb | 2 (12.5%) | 2 (14.3%) | 1 | 2 (14.3%) | 2 (12.5%) | 1 |
|  | IV | 14 (87.5%) | 12 (85.7%) |  | 12 (85.7%) | 14 (87.5%) |  |
| Histology | Adenocarcinoma | 9 (56.2%) | 7 (50%) | 1 | 8 (57.1%) | 8 (50%) | 0.98 |
|  | Squamous carcinoma | 7 (43.8%) | 7 (50%) |  | 6 (42.9%) | 8 (50%) |  |

**Table S4**. Baseline clinical characteristics of 30 patients with NSCLC stratified into 4 subgroups according to PD-L1 tumor proportion score and CD8+ T cell using a cutoff value of 1%.

| Characteristics | | PD-L1 TPS ≥1% | | PD-L1 TPS < 1% | | *p* |
| --- | --- | --- | --- | --- | --- | --- |
|  |  | CD8≥1% (n=8) | CD8<1% (n=8) | CD8≥1% (n=6) | CD8<1% (n=8) |  |
|  |  | PD-L1+/CD8+ | PD-L1+/CD8- | PD-L1-/CD8+ | PD-L1-/CD8- |  |
| Sex | Female | 2 (25%) | 3 (37.5%) | 1 (20%) | 2 (25%) | 0.847 |
|  | Male | 6 (75%) | 5 (62.5%) | 5 (80%) | 6 (75%) |  |
| Age | <56 years | 4 (50%) | 4 (50%) | 4 (66.7%) | 3 (37.5%) | 0.760 |
|  | ≥56 years | 4 (50%) | 4 (50%) | 2 (33.3%) | 5 (62.5%) |  |
| Smoking history | Former smoker | 6 (75%) | 3 (37.5%) | 3 (50%) | 4 (50%) | 0.497 |
|  | Never smoker | 2 (25%) | 5 (62.5%) | 3 (50%) | 4 (50%) |  |
| ECOG PS score | 0-1 | 8 (100%) | 8 (100%) | 6 (100%) | 8 (100%) | 1 |
|  | ≥2 | 0 | 0 | 0 | 0 |  |
| Stage | IIIa/IIIb | 1 (12.5%) | 1 (12.5%) | 1 (20%) | 1 (12.5%) | 0.994 |
|  | IV | 7 (87.5%) | 7 (87.5%) | 5 (80%) | 7 (87.5%) |  |
| Histology | Adenocarcinoma | 4 (50%) | 4 (50%) | 3 (50%) | 5 (62.5%) | 0.946 |
|  | Squamous carcinoma | 4 (50%) | 4 (50%) | 3 (50%) | 3 (37.5%) |  |

**Figure S1**. The median ratio of PD-L1 expressing circulating tumor cells (CTC).


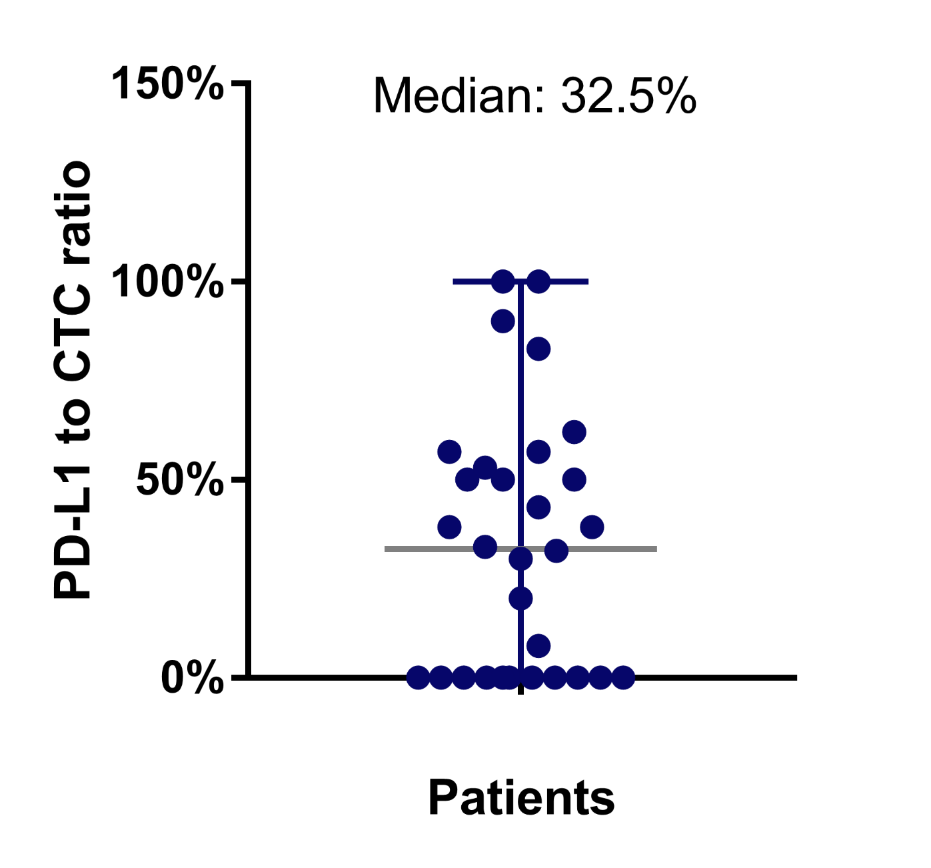

Supplement: Supplementary file 1 — Additional file 1: Table S1. Treatment-related adverse events observed from the study cohort (N = 30). Table S2. Baseline clinical characteristics of 30 patients with NSCLC stratified according to their PD-L1 expression in circulating tumor cells (CTC PD-L1) ratio using a cutoff value of 32.5%. Table S3. Baseline clinical characteristics of 30 patients with NSCLC stratified according to a cutoff value of 1% for CD8 T cell and tissue-based PD-L1 expression. Table S4. Baseline clinical characteristics of 30 patients with NSCLC stratified into 4 subgroups according to PD-L1 tumor proportion score and CD8+ T cell using a cutoff value of 1%. Fig. S1. The median ratio of PD-L1 expressing circulating tumor cells (CTC). [file 12885_2022_10045_MOESM1_ESM.docx]
